# 

**Published:** 1920-03-27

**Authors:** 


March 27, 1920] [Price Twopence

### The Hospital

The Workers' Newspaper of Administrative Medicine and Institutional Life, Administration, National Insurance and Health.

### CONTENTS.

**King Edward's Fund and the London Voluntary Hospitals** ... ... ... ... ... ... 603

**Safe Milk** ... ... ... ... ... ... ... 604

**Hospital and Institutional News** ... ... ... 605

Infant Mortality—Representative Medicine—The "Pall Mall" Panacea—The International Red Cross—The School Doctor on Leeds Children—The Optimist and the Pessimist—The Leper Problem in India—Cocaine Habit in Bengal—Lord Knutsford on Co-ordination—A Cheerful Lesson from Belfast—Overcrowding in Furniture—No Nonsense at Newport!—An Octogenarian's Generosity—Women in Hospital Life—Personal Service Penalised—Finance and Personal Service—Clayton Hospital's New Secretary—A Hospital League at Torquay—Registrars of Births and Deaths and the Increased Cost of Living—Acton Cottage Hospital Extensions—Home for Last-Stage Consumptives—A Test of Municipal Hospital Schemes—Increase of Maternity Cases—Proposed County Hospital for Rutland—Cinema in Hospital—Should District Medical Officers be Abolished?—This Week's Drug Market.

**Radiography in Hepatic Conditions** ... ... 609

A Valuable Aid to Diagnosis.

**Industrial Medicine** ... ... ... ... ... ... 610

**Chronic Intestinal Stasis** ... ... ... ... 611

New Discoveries in this Disease.

### CONTENTS

**The Severer Forms of Laryngitis** ... ... 613

II. Early Diagnosis and Treatment (*continued*).

**The Ministry and Unregistered Dentists** ... ... 614

**Hospital Construction: The New Era** ... ... 615

The Sanitary Blocks of Hospitals.

**Scrapping the Trained Nurse.—II.** ... ... 616

Expert Opinion.

**The Matrons' and Sisters' Department** ... ... 617

The Universities and the Nursing Profession.

**Round the Hospitals** ... ... ... ... ... ... 617

**The Public Well-Being** ... ... ... ... ... 621

Cleansing the Air—Care of the Unborn—Humans or Dogs?—Adopted Children—Rate Aid for Unmarried Mothers—International Health Work.

**Present Position of the London Voluntary Hospitals** 625

Statement by King Edward's Hospital Fund.

**Annual Meetings of Hospitals** ... ... . 626

**Contributions from Patients** ... ... ... 627

**The Editor's Letter-Box** ... ... ... ... 628

**Parliament and the Professions** ... ... ... v

**The College of Nursing (Limited by Guarantee)** ... v

**Matrons' and Sisters' Appointments** ... ... ... v

**The Institutional Worker** ... ... ... ... 1

